# Causes of varied internal echo patterns in ultrasound imaging of breast lymphoma

**DOI:** 10.1007/s12282-025-01820-5

**Published:** 2026-04-07

**Authors:** Yumi Kokubu, Tomo Osako, Takehiko Sakai, Yui Tomita, Chieko Kato, Yuri Maruoka, Masashi Akiya, Chikako Takahata, Takayuki Ueno, Kiyoshi Matsueda

**Affiliations:** 1 Department of Ultrasound/IVR, Cancer Institute Hospital, Japanese Foundation for Cancer Research, Tokyo, Japan; 2 Department of Diagnostic Imaging, Cancer Institute Hospital, Japanese Foundation for Cancer Research, Tokyo, Japan; 3 Division of Pathology, Cancer Institute, Japanese Foundation for Cancer Research, Tokyo, Japan; 4 Department of Pathology, Cancer Institute Hospital, Japanese Foundation for Cancer Research, Tokyo, Japan; 5 Department of Surgical Oncology, Breast Oncology Center, Cancer Institute Hospital, Japanese Foundation for Cancer Research, Tokyo, Japan; 6 Department of Ultrasound, Cancer Institute Hospital, Japanese Foundation for Cancer Research, Tokyo, Japan

**Keywords:** Breast lymphoma, Breast ultrasound, High-resolution ultrasound, Internal echo pattern, Breast biopsy

## Abstract

**Background:**

Ultrasound (US) imaging of breast lymphoma often reveals a variety of internal echo patterns, making differentiation from breast cancer challenging. This study aimed to correlate pathological findings with US imaging features of breast lymphoma to enhance biopsy accuracy and diagnostic precision.

**Materials and methods:**

We retrospectively analyzed 37 lesions (36 cases) with available US images and needle biopsy pathology specimens from breast lymphoma patients at our hospital between 2010 and 2021. The area ratios of tumor cells, mammary gland tissue, and adipose tissue in pathology specimens were compared with internal echo patterns on US. Additionally, we assessed US images during the healing process in cases with complete resolution following treatment.

**Results:**

US identified 34 of 37 lesions (92%) as masses. Of these, 23 (68%) exhibited mixed internal echoes, while 11 (32%) were hypoechoic. Among the mixed echo lesions, 9 (27%) displayed a mixed hyper-to-hypoechoic pattern, and 14 (41%) showed a mixed iso-to-hypoechoic pattern. Pathological analysis revealed that the tumor cell median area ratio was highest in hypoechoic masses, mammary gland tissue median ratio was highest in mixed iso-to-hypoechoic masses, and adipose tissue median ratio was highest in mixed hyper-to-hypoechoic masses. During healing, the mass changed to non-mass abnormalities in 10 cases (77%) and decreased in size while retaining its shape in 3 cases (23%).

**Conclusion:**

The internal echo patterns of breast lymphoma varied depending on three factors: the proportion of tumor cells; the presence of regions with low tumor cell density; and adipose tissue infiltration. Hypoechoic areas likely reflected tumor cells, isoechoic areas mammary gland tissue, and hyperechoic areas adipose tissue infiltration. These findings suggest that biopsy from hypoechoic regions is recommended for accurate diagnosis.

## Introduction

Breast lymphoma is a rare disease accounting for 0.04% to 0.53% of malignant breast tumors [1–3].” Breast lymphoma can grow rapidly, necessitating prompt and accurate diagnosis.

The treatment of breast lymphoma is mainly chemotherapy, while surgery may be the first choice for breast cancer. Despite the importance of distinguishing between the two, it is currently difficult due to the nonspecific images of breast lymphoma, which are similar to those of breast cancer [4–6].

While breast lymphoma has been reported to show a variety of internal echo patterns on ultrasound (US) [6–10], there are no reports in which the cause of this variation has been examined in detail. Since breast lymphoma is not surgically resected, it is not possible to compare US images with pathological findings. However, US images can be compared with pathological tissue findings obtained by needle biopsy.

In recent years, high-resolution US devices have made it possible to observe the normal structure and lesions of the mammary gland in greater detail than before [11–13]. Slight differences in the internal echo level of lesions can be depicted, and lesions with truly homogeneous internal echoes are rare. It is becoming possible to obtain image findings that more closely reflect pathological macro-images. The internal echo pattern is determined by the balance between reflection and transmission/attenuation of ultrasound waves. In general, hypo-echogenicity within a mass occurs when it is composed of homogeneous tumor cells, whereas hyper-echogenicity arises when components with differing acoustic impedances are intermixed. In general, internal echo patterns of breast cancer vary according to the components of the lesion such as tumor cells, adipose tissue, fibrous tissue, mucinous components, cystic components, and necrotic tissue. On the other hand, since lymphoma lesions are mainly composed of tumor cells and mammary gland tissue, and adipose tissue; therefore, increased echogenicity is considered to result from the intermixture of these components. Thus, tumor cell density may be associated with internal echo patterns. If the association between tumor cell density and internal echo patterns within the lesions is clarified, this would lead to more accurate biopsies and diagnoses. Furthermore, evaluating US changes during the healing process in cases that achieved complete remission may provide greater insight into lesion composition. In this study, we divided internal echo patterns into hypoechoic lesions and lesions with mixed echo levels. We then further divided the lesions with mixed echo levels into mixed hyper-to-hypoechoic lesions and mixed iso-to-hypoechoic lesions for evaluation.

We aimed to clarify the cause of internal echo patterns in breast lymphoma by evaluating the internal echo levels of lesions and comparing them with histopathological findings from needle biopsy specimens.

## Materials and methods

### Patients

From 2010 to 2021, 12,522 lesions were diagnosed as malignant based on pathological diagnoses of breast needle biopsy. Of these, 45 lesions from 44 cases (0.4%) were diagnosed with breast lymphoma. In this study, US images and needle biopsy pathology specimens from 36 cases (37 lesions) were reviewed. In addition, when multiple lesions were present in the same breast, only the mass on which the biopsy was performed was evaluated. This retrospective study identified all cases with both available ultrasound images and histopathological specimens by searching the pathology database. This study was approved by the ethics committee of the institution, and the committee determined that informed consent could be simplified. [2022-GB-019].

### Equipment

For US imaging, we used the Aplio 500, the AplioXG (Canon Medical Systems, Tokyo, Japan), and the LOGIQ E9 (GE Healthcare, Milwaukee, WI, USA), while the probes used were all linear transducers of 10 MHz or higher.

### Methods

The following four items were evaluated:

1. The US images of lesions
2. The median area ratios of tumor cells, mammary gland tissue, and adipose tissue in needle biopsy pathology specimens
3. The presence or absence of regions with low tumor cell density in needle biopsy pathology specimens
4. US images during the healing process in cases that achieved complete remission following treatment

1. Evaluation of the US images of lesions (Fig. 1)
  **Fig. 1:**
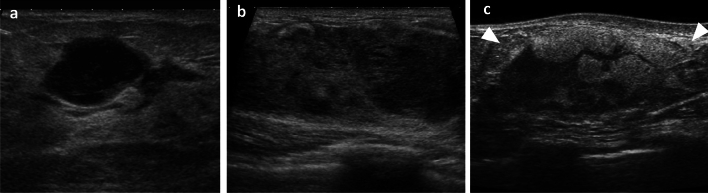
Examples of internal echo pattern evaluations of masses. **a** Hypoechoic mass. **b** Mixed iso-to-hypoechoic mass. **c** Mixed hyper-to-hypoechoic mass
  In this study, all pre-biopsy ultrasound images were reviewed and retrospectively re-evaluated on a case-by-case basis. US images of lesions were evaluated in accordance with the Breast Ultrasound Guidelines [14] of JABTS. Lesions were classified into masses and non-mass abnormalities. Masses were evaluated for shape, margin, posterior echo, and internal echo pattern. Internal echo patterns of masses were classified into hypoechoic and mixed echo. Masses with a mixed echo were further classified into mixed hyper-to-hypoechoic and mixed iso-to-hypoechoic masses, and the US findings of each were evaluated.
2. Evaluation of the median area ratios of tumor cells, mammary gland tissue, and adipose tissue in needle biopsy pathology specimens (Fig. 2).
  **Fig. 2:**
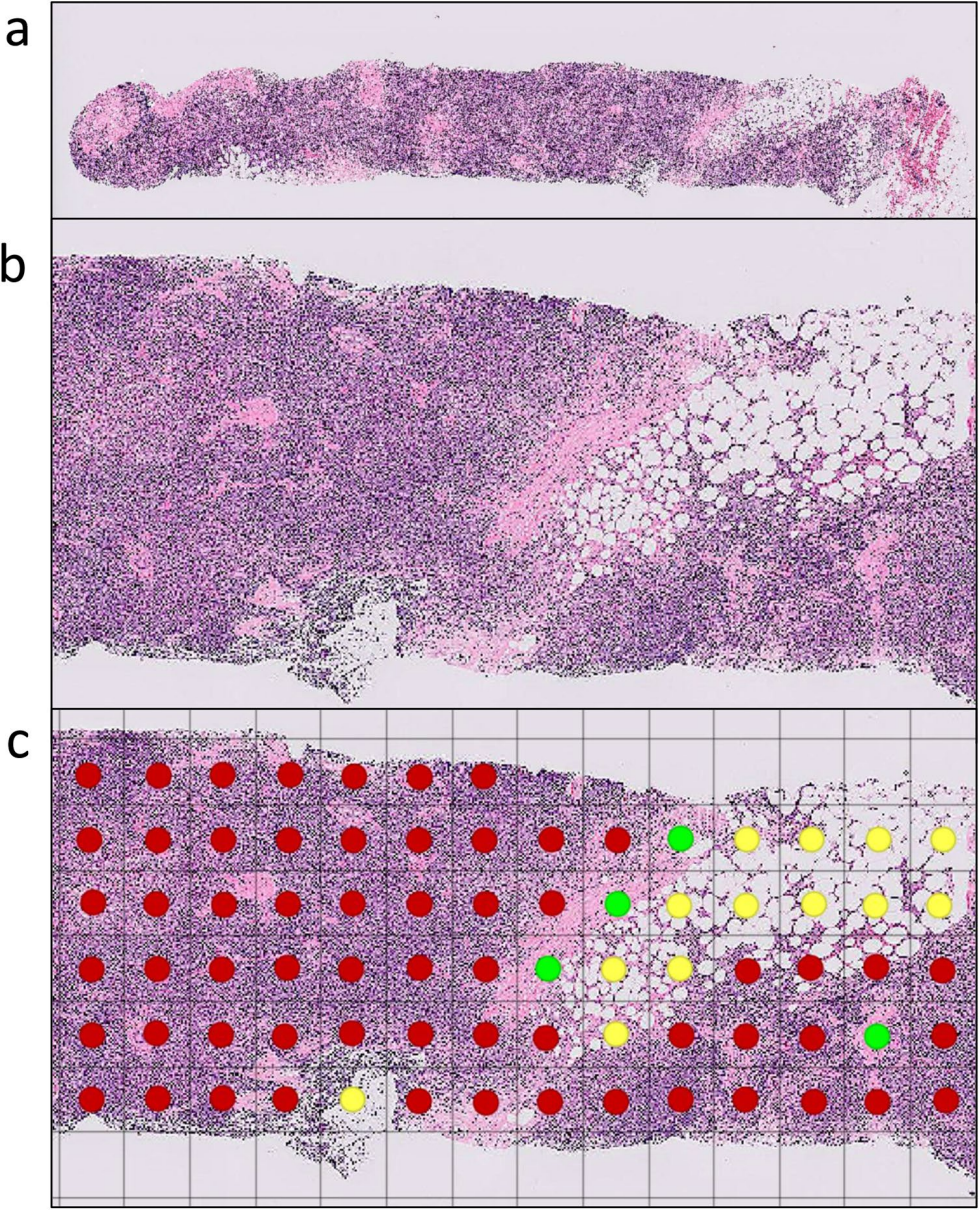
Examples of evaluating the area ratios of tumor cells, mammary gland tissue, and adipose tissue in needle biopsy pathology specimens. **a** Hematoxylin and eosin (H&E)–stained core needle biopsy specimen. **b** Partially magnified view of the specimen shown in (a). **c** The histological image shown in (b) is overlaid with a 250 µm × 250 µm grid. Each grid square was then evaluated semi-quantitatively by assigning one predominant component (tumor cells, mammary gland tissue, or adipose tissue). In this figure, tumor cells are annotated in red, mammary gland tissue in green, and adipose tissue in yellow for visualization
  The area ratios of tumor cells, mammary gland tissue, and adipose tissue in hematoxylin and eosin (H&E)-stained specimens obtained from needle biopsy were quantitatively evaluated. When multiple cores were obtained from a lesion, the area ratios were calculated from the total measurable tissue across all specimens. All histological images were imported into QuPath (version 0.6.0) and overlaid with a 250 µm × 250 µm grid. Each grid square was then evaluated semi-quantitatively by assigning one predominant component (tumor cells, mammary gland tissue, or adipose tissue). This approach represents semi-quantitative annotation rather than pixel-wise segmentation, as one dominant tissue type was allocated per grid unit without delineating precise structural boundaries. The area ratios for each lesion were subsequently calculated, and the median values with interquartile ranges (IQR) were determined according to internal echo patterns. After all pathological specimens were reviewed under the supervision of a board-certified pathologist, the annotations were performed by a board-certified diagnostic radiologist.
3. Evaluation of the presence or absence of regions with low tumor cell density in needle biopsy pathology specimens (Fig. 3).
  **Fig. 3:**
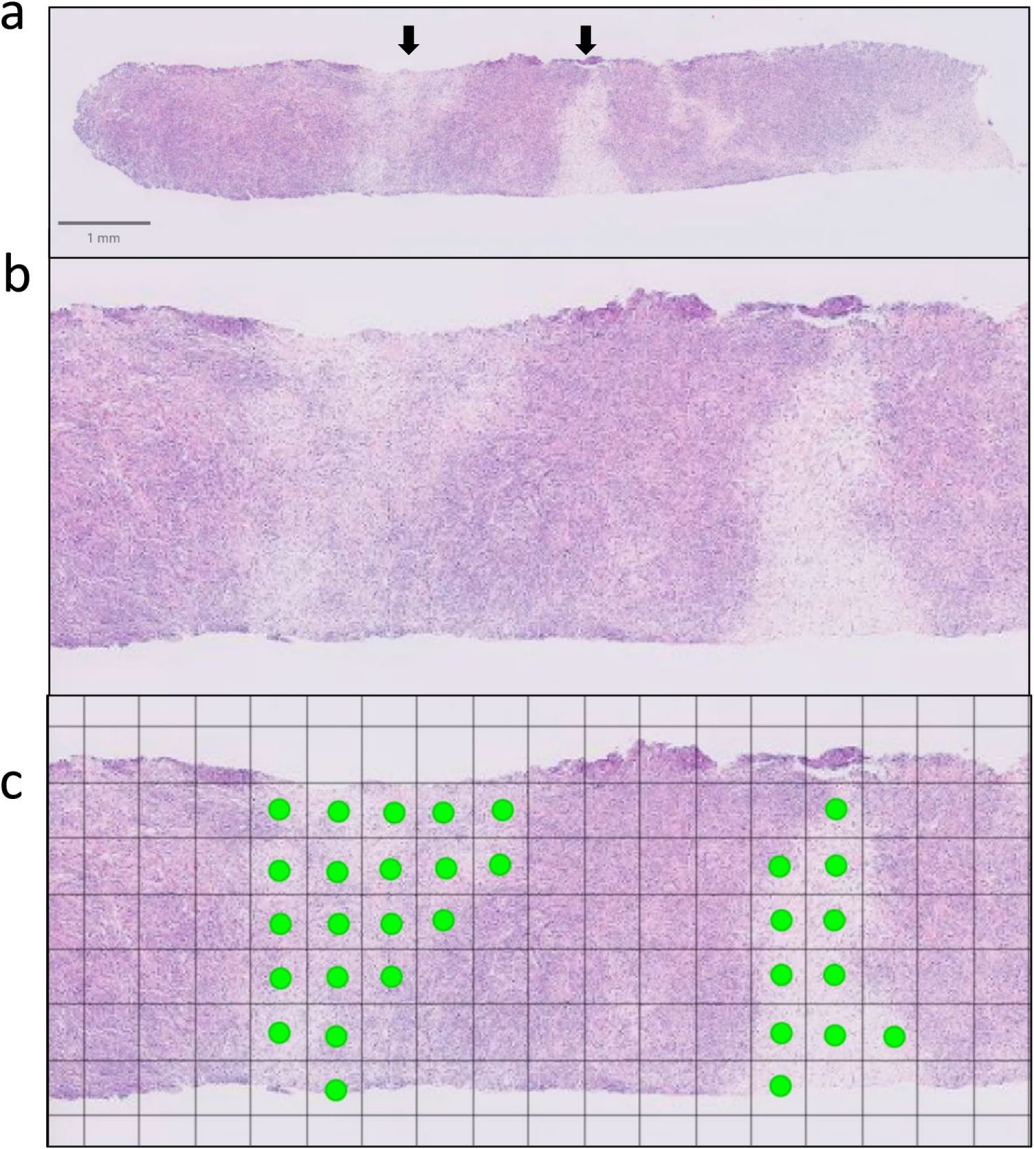
Evaluation of regions with low tumor cell density in needle biopsy pathology specimens. **a** Hematoxylin and eosin (H&E)–stained core needle biopsy specimen. **b** Partially magnified view of the specimen shown in (a). **c** The histological image shown in (b) is overlaid with a 250 µm × 250 µm grid. In this specimen, two regions with low tumor cell density are identified, each forming a cluster occupying eight or more squares on the grid
  In hematoxylin and eosin–stained needle biopsy specimens unequivocally obtained from within the mass, some cases demonstrated regions characterized by low tumor cell density. These regions were considered to be potentially related to the internal echo characteristics on US. Therefore, when regions with low tumor cell density were found to be aggregated within ≥ 8 squares of the 250 µm × 250 µm grid, the lesion was classified as having low-density regions, and their presence or absence was recorded. In addition, the frequency of lesions containing regions with low tumor cell density was compared according to the internal echo patterns.
4. US images during the healing process in cases that achieved complete remission following treatment.
  We evaluated how lesions disappeared for cases where US images were obtained during the course of treatment. The frequency of each pattern was evaluated: those that changed from mass to non-mass lesions, those that changed from non-mass to mass lesions, and those that shrank while remaining in the form of mass or non-mass lesion.

### Statistical analysis

Continuous variables were summarized as the median and interquartile range (IQR). The sizes of mass and non-mass lesions were evaluated using the median and IQR, as were the area ratios of tumor cells, mammary gland tissue, and adipose tissue according to internal echo patterns. All statistical analyses were conducted using R (version 4.5.2).

## Results

### Patient characteristics

All 36 cases were female, with an average age of 58.3 years (range: 22–83 years); Of these, 30 cases were primary breast lymphoma (PBL), and 6 cases were secondary breast lymphoma (SBL). All 36 cases had received no prior treatment for breast lesions. One of the 36 patients had lesions on both breasts, resulting in a total of 37 lesions were targeted. The disease types were diffuse large B-cell lymphoma (DLBCL) in 30 patients (81%), mucosa-associated lymphoid tissue (MALT) lymphoma in 4 patients (11%), follicular lymphoma in 1 patient with bilateral breast lesions (5%), and T-cell lymphoma in 1 patient (3%).

1. US evaluation of lesions (Tables 1, 2)
  **Table 1:** Breakdown of the masses vs. non-mass abnormalities in 37 lesions of breast lymphoma

Characteristics	Total	DLBCL	MALT	Follicular	Tcell
	n (%)	n (%)	n (%)	n (%)	n (%)
Mass	34 (92)	29 (97)	2 (50)	2 (100)	1 (100)
Non-mass abnormality	3 ( 8)	1 ( 3)	2 (50)	0 ( 0)	0 ( 0)

*DLBCL*diffuse large B-cell lymphoma,*MALT*mucosa-associated lymphoid tissue
  **Table 2:** US findings of 34 masses in breast lymphoma

	Total	DLBCL	MALT	follicular	Tcell
	n (%)	n (%)	n (%)	n (%)	n (%)
	34 (100)	29 (100)	2 (100)	2 (100)	1 (100)
*Shape*					
Round/oval	16 (47)	15 (52)	0 ( 0)	0 ( 0)	1 (100)
Lobulated	13 (38)	10 (34)	2 (100)	1 (50)	0 ( 0)
Irregular	5 (15)	4 (14)	0 ( 0)	1 (50)	0 ( 0)
*Margin*					
Circumscribed	7 (20)	6 (21)	1 (50)	0 ( 0)	0 ( 0)
Microlobulated	23 (68)	19 (65)	1 (50)	2 (100)	1 (100)
Indistinct	4 (12)	4 (14)	0 ( 0)	0 ( 0)	0 ( 0)
*Echo pattern*					
Hypoechoic	11 (32)	7 (24)	2 (100)	2 (100)	0 ( 0)
Mixed hyper-to-hypoechoic	23 (68)	22 (76)	0 ( 0)	0 ( 0)	1 (100)
Iso-to-hypoechoic	14 (41)	13 (45)	0 (0)	0 (0)	1 (100)
Hyper-to-hypoechoic	9 (27)	9 (31)	0 (0)	0 (0)	0 (0)
*Posterior features*					
Enhancement	27 (79)	25 (86)	1 (50)	0 ( 0)	1 (100)
None	7 (21)	4 (14)	1 (50)	2 (100)	0 ( 0)
Shadowing	0 ( 0)	0 ( 0)	0 ( 0)	0 ( 0)	0 ( 0)

*DLBCL* diffuse large B-cell lymphoma, *MALT* mucosa-associated lymphoid tissue
  US findings for 37 lesions showed 34 mass lesions (92%) and 3 non-mass abnormalities (8%). There were 28 cases with a single mass and 6 cases with multiple masses. For those with multiple masses, only the site where the biopsy was performed was evaluated. The median (IQR) size of the 34 mass lesions was 40.5 mm (24–64 mm), and that of the three non-mass abnormalities was 48 mm (35.5–54 mm).
  Of the 34 mass lesions, 16 (47%) were round or oval in shape, 13 (38%) were lobular in shape, and 5 (15%) were irregular in shape. Mass margins were circumscribed in 7 lesions (20%), well-defined and rough in 23 lesions (68%), and indistinct in 4 lesions (12%). Posterior echoes showed accentuation in 27 lesions (79%) and no posterior features in 7 lesions (21%), with no lesions exhibiting attenuation of the posterior echo. Internal echo patterns indicated that 23 mass lesions (68%) had mixed echo patterns, while 11 mass lesions (32%) had hypoechoic patterns. Among the masses with mixed echo patterns, 9 lesions (27%) were classified as mixed hyper-to-hypoechoic, while 14 lesions (41%) were classified as mixed iso-to-hypoechoic. No masses with echogenic foci due to calcification were observed. In addition, there were no lesions with cystic portions or architectural distortion.
  All three lesions appeared as hypoechoic areas on US. Two of these lesions (one MALT lymphoma and one DLBCL) presented with a geographic hypoechoic area, while the remaining MALT lymphoma lesion showed a mottled hypoechoic area.
2. Evaluation of the median area ratios of tumor cells, mammary gland tissues, and adipose tissues in needle biopsy pathology specimens (Table 3).
  **Table 3:** Area Ratios of Tumor Cells, Mammary Gland Tissue, and Adipose Tissue, and Presence of regions with low tumor cell density in Pathological Biopsy Specimens According to Internal Echo Pattern *n* = 31

Hypoechoic mass *n* = 11					Iso-to-hypoechoic mass *n* = 12					Hyper-to-hypoechoic mass *n* = 8				
Case No	Area ratio (%)			Regions with low tumor cell density	Case No	Area ratio (%)			Regions with low tumor cell density	Case No	Area ratio (%)			Regions with low tumor cell density
	Tumor cell	Mammary gland tissue	Adipose tissue			Tumor cell	mammary gland tissue	Adipose tissue			Tumor cell	Mammary gland tissue	Adipose tissue	
1	97	3	0	–	12	90	10	0	–	24	91	1	8	–
2	97	3	0	–	13	88	12	0	+	25	91	3	6	+
3	97	3	0	–	14	84	16	0	+	26	88	12	0	–
4	96	4	0	–	15	81	19	0	–	27	82	13	5	+
5	95	5	0	–	16	74	26	0	+	28	81	9	10	–
6	94	2	4	–	17	72	23	5	+	29	71	13	16	–
7	92	7	1	–	18	71	29	0	+	30	67	0	33	+
8	88	12	0	–	19	58	42	0	+	31	12	35	53	+
9	83	12	5	–	20	56	42	2	+					
10	75	18	7	+	21	46	49	5	+					
11	63	37	0	+	22	45	43	12	+					
					23	29	66	5	+					
Median [IQR]	94 [86–97]	5 [3–12]	0 [0–3]		Median [IQR]	72 [54–82]	28 [18–42]	0 [0–5]		Median [IQR]	82 [70–89]	11 [3–13]	9 [6–20]	
total(%)				2 (18)	total(%)				10 (83)	total(%)				4 (50)

**Fig. 5:**
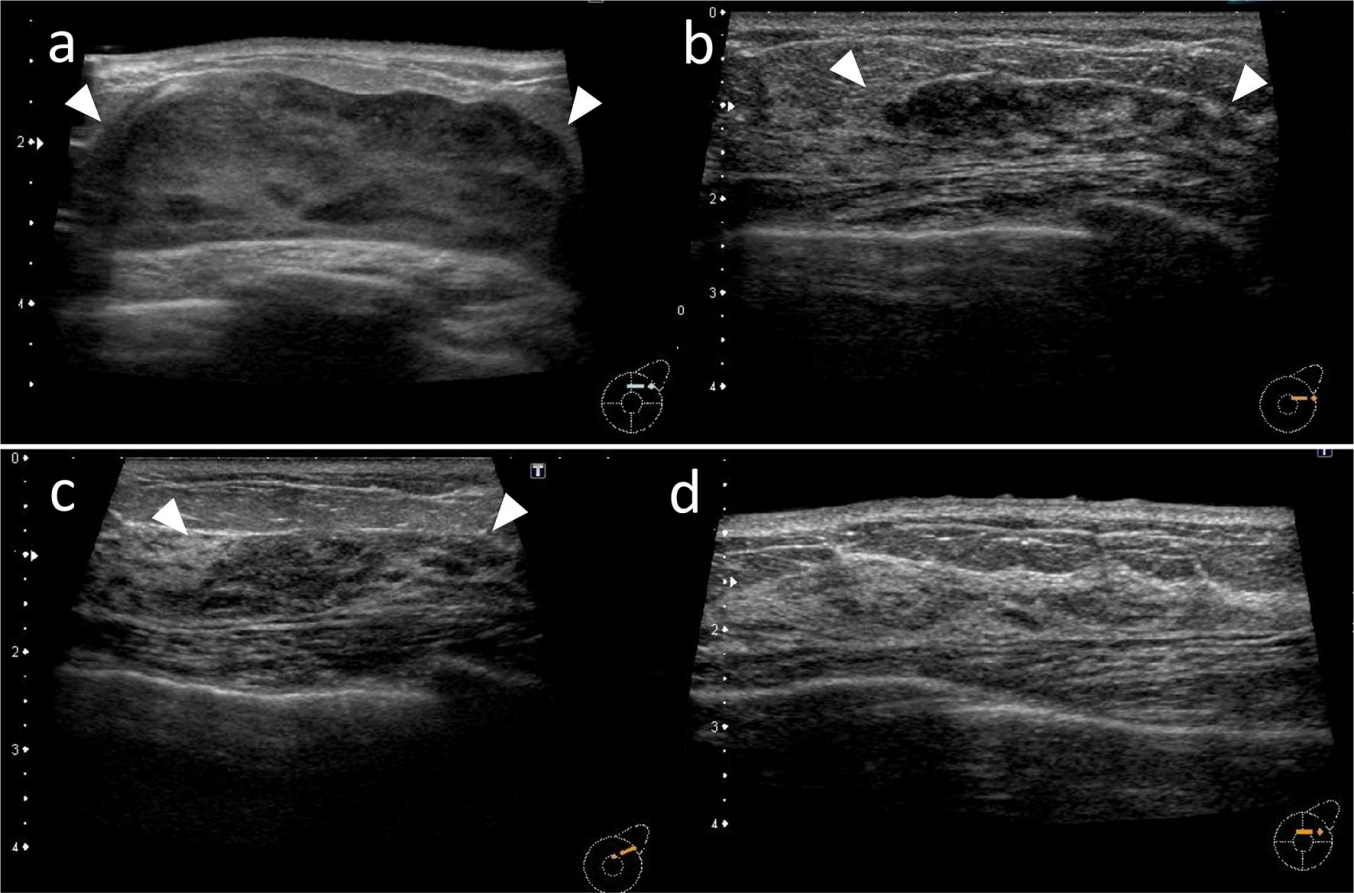
Female in her 50 s with DLBCL (Case No. 17): US images of a case of complete remission case with chemotherapy. **a** At symptom onset. Masses are seen as huge masses occupying left breast (arrowhead). The size of the mass is 75 × 70 × 21 mm and the internal echo pattern exhibits a mixed iso-to-hypoechoic pattern. **b** 7 months after the start of chemotherapy. The boundary of the mass is indistinct and has changed to a hypoechoic area (arrowhead). Part of the hypoechoic area is lumpy. **c** 8 months after the start of chemotherapy. The lesion is observed as a hypoechoic area (arrowhead). Echo levels have slightly increased throughout. **d** 12 months after the start of chemotherapy. The hypoechoic area has disappeared, and no abnormalities are detectable at the site where the lesion was previously located
  28 cases were completely resolved by treatment (28 lesions: 25 mass lesions, 3 non-mass abnormalities). US images of the healing process of 13 cases (11 mass lesions, 2 non-mass abnormalities) in which disappearance of lesions was confirmed were evaluated.
  Among the 11 mass lesions, 9 lesions (82%) changed to non-mass abnormalities during the healing process, while 2 lesions (18%) shrank centripetally while maintaining their shape as a mass. Two of the non-mass abnormalities (Case No. 36, 37) confirmed by US imaging of the healing process were also recognized as non-mass abnormalities during the healing process. Non-mass abnormalities did not change into masses during the healing process.

Median [IQR]: median [interquartile range]
  The area ratios of tumor cells, mammary gland tissue, and adipose tissue in the histopathology of biopsy specimens was evaluated. However, three lesions in which the boundary between the mass and the peripheral tissue in the pathology specimen could not be evaluated were excluded.
  The median (IQR) area ratios of tumor cells, mammary gland tissue, and adipose tissue in needle biopsy specimens from hypoechoic masses were 94% (86–97%), 5% (3–12%), and 0% (0–3%), respectively. In mixed iso-to-hypoechoic masses, the ratios were 72% (54–82%), 28% (18–42%), and 0% (0–5%). In mixed hyper-to-isoechoic masses, the ratios were 82% (70–89%), 11% (3–13%), and 9% (6–20%).
  Among tumor cells with internal echo patterns of hypoechoic, mixed iso-to-hypoechoic, and mixed hyper-to-hypoechoic, the median area ratio of tumor cells was highest in hypoechoic masses. The median area ratio of mammary gland tissues was highest in mixed iso-to-hypoechoic masses. The median area ratio of adipose tissue was highest in mixed hyper-to-hypoechoic masses. In addition, in pathological specimens of mixed hyper-to-hypoechoic masses, tumor cells infiltrating into adipose tissues were confirmed in 7 of 8 lesions (88%).
3. Evaluation of the presence or absence of regions with low tumor cell density in needle biopsy pathology specimens (Table 3).
  The proportion of regions with low tumor cell density in the pathological tissue of biopsy specimens was evaluated. The numbers of lesions containing regions with low tumor cell density at each internal echo pattern were as follows: 4 lesions (50%) in the mixed hyper-to-hypoechoic pattern, 10 lesions (83%) in the mixed iso-to-hypoechoic pattern, and 2 lesions (18%) in the hypoechoic pattern. The regions with low tumor cell density were most common in mixed iso-to-hypoechoic masses and least common in hypoechoic masses.
4. US images during the healing process in cases that achieved complete remission following treatment (Figs. 4, 5).
  **Fig. 4:**
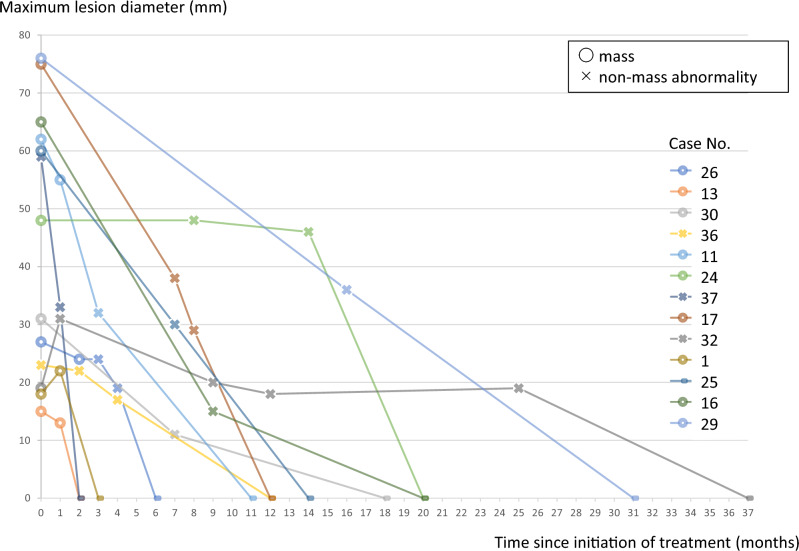
Change in the maximum diameter during the healing process in a completely resolved cases. This diagram illustrates the change in the maximum diameter over time in 13 lesions in which the US image of the healing process was confirmed. The shape of the lesion is shown as a circle (〇) for masses and a cross ( ×) for non-mass abnormalities. Many of the masses had changed to non-mass abnormalities at an early stage after the start of treatment

## Discussion

It has been reported that US imaging of breast lymphoma is characterized by a round or oval shape, with internal echo patterns exhibiting hypoechoic or a variety of echo level mixes. 92% of the lesions in this study exhibited mass lesions, while only 8% were non-mass abnormalities. The majority of the masses were round, oval, or lobular in shape. The boundaries were well-defined and rough. Posterior echoes indicated accentuation in 79% of cases and no posterior features in 21%, with no attenuation observed. This was thought to be due to the absence of fibrous component proliferation, which is the main cause of posterior echo attenuation. Similar findings with no posterior echo attenuation have been reported in the past [7, 8, 10].

Approximately 1/3 of the internal echo patterns exhibited hypoechoic, while the remaining 2/3 were a variety of echo pattern mixes. In this study, the internal echo patterns of masses were more likely to exhibit a variety of echo pattern mixes than hypoechoic. In contrast, previous reports found the frequency of hypoechoic lesions was higher than that of lesions with a variety of echo pattern mixes [5, 7–9, 15]. One possible reason for these differences is in the utilization of different equipment. In this study, a high-resolution device and a high-frequency probe made it possible to study internal echo patterns in three ways: hypoechoic, mixed iso-to-hypoechoic, and mixed hyper-to-hypoechoic. Mixed iso-to-hypoechoic lesions in this study may have been classified as heterogeneous hypoechoic masses in previous reports.

Mixed hyper-to-hypoechoic masses tended to have a higher median area ratio of adipose tissues than hypoechoic or mixed iso-to-hypoechoic masses. On the other hand, in mixed iso-to-hypoechoic masses, the median area ratio of mammary gland tissues was higher than that of other masses, with hypoechoic masses tending to have a higher median area ratio of tumor cells than that of other masses. Lymphoma lesions have a very high tumor cell density, and areas with minimal non-tumor tissue appear hypoechoic on US images. On the other hand, if the background mammary gland is fatty or if subcutaneous adipose tissue is infiltrated by tumor cells, the mixed adipose tissue and tumor cells cause ultrasound backscattering, resulting in hyperechoic images. Additionally, if tumor cells are mixed within the interstitial tissue of the mammary gland, they are believed to produce isoechoic images. For this reason, we considered that the hyperechoic part reflected infiltration into adipose tissue, the iso-echoic part reflected mammary gland tissue, and the hypoechoic part reflected tumor cells.

While some reports attribute hyper-echogenicity in lymphoma masses to tumor cell infiltration into adipose tissue [16–18], other reports suggest that high tumor cell density is the cause [5]. In this study, the cause of iso-echoes can be inferred by dividing the mixed internal echo patterns into mixed hyper-to-hypoechoic and mixed iso-to-hypoechoic areas.

A region with low tumor cell density was observed in more than 80% of mixed iso-to-hypoechoic and 50% of mixed hyper-to-hypoechoic lesions. A region with low tumor cell density was considered a factor indicating a variety of internal echo patterns. Lymphoma lesions are formed by cancerized lymphocytes, and the proliferation of fibrous tissue that is often observed in breast cancer is almost never observed. Therefore, the lesions themselves are formed by cancerized lymphocytes and normal mammary gland tissue. US findings of the lesions vary depending on the density of tumor cells and the component of the background mammary gland.

Calcification was not observed in the biopsy pathology specimens of the lesions targeted. In addition, no hyperechoic foci suggesting calcification were observed in the target lesion even in US images. While there are similar reports of no calcification in lymphoma [1, 3, 6–9, 19], there are also reports where calcification has been observed [4, 10]. In addition, no spicula or architectural distortion was observed at the margins of the masses, similar to previous reports [1, 8, 19].

In lymphoma lesions, it was sometimes difficult to distinguish between masses and non-mass abnormalities. Fibrous capsules, which can be observed in breast cancer, are not formed around lymphoma masses. Rather, these masses are composed solely of tumor cells. When the tumor cell density at the margin of the mass was low, it was thought that the boundary with the normal structure was blurred.

In this study, a hypoechoic area in the mammary gland was observed in the three patients with non-mass abnormalities. In this study, the three patients with non-mass abnormalities exhibited a hypoechoic area in the mammary gland. Because of their low tumor cell density throughout the lesion, they were not recognized as masses and are thought to have exhibited an image of non-mass abnormalities. When an increase in tumor cell density occurred, it was thought that their hypoechoic area could easily change into a mass. Interestingly, we found that 2 of the 3 non-mass abnormalities were MALT lymphomas. Because MALT lymphoma has a slow progression [20], it may have been diagnosed prior to the formation of masses.

In the evaluation of images during the course of treatment, there were more masses that changed to non-mass abnormalities compared to those that shrank while maintaining the shape of the mass, accounting for 82% of the total. These lesions were thought to exhibit an image of non-mass abnormalities due to decreased tumor cell density.

## Limitations

There are several limitations of this study. Specifically, it was a single-center study. Although biopsy specimens represent only a portion of the lesion and may not fully reflect the overall composition, the internal echo pattern typically shows a mixture of echoes rather than an extreme difference between the central and peripheral areas of the mass. By evaluating multiple biopsy specimens obtained from each lesion across multiple cases, we anticipated that trends corresponding to different patterns of internal echoes would emerge, which provided the rationale for conducting this study. In addition, although the area ratios measured from pathological specimens were calculated using a 250 µm × 250 µm grid, the assessment relied on visual estimation, which should be acknowledged as a limitation.

Furthermore, while there were 30 lesions of DLBCL, other types were much less common, with only 4 cases of MALT lymphoma, 2 cases of follicular lymphoma, and 1 case of T-cell lymphoma. This number was insufficient to evaluate differences in US findings among disease subtypes. For lymphoma subtypes other than DLBCL, further analyses will be warranted as more cases become available in the future.

## Conclusion

Approximately 2/3 of the internal echo patterns in the mass of breast lymphoma were mixed hyper-to-hypoechoic or mixed iso-to-hypoechoic, while approximately 1/3 were hypoechoic. It was thought that internal echo patterns varied depending on three factors: the proportion of tumor cells; the presence of regions with low tumor cell density; and adipose tissue infiltration. Hypoechoic areas were thought to correspond to tumor cells, iso-echoes areas were thought to correspond to mammary gland tissue, and hyperechoic areas were thought to correspond to tumor cell infiltration into adjacent adipose tissue. From the above, understanding the underlying basis of internal echo patterns in lymphoma makes it evident that performing biopsy from the hypoechoic regions is essential. Obtaining specimens from areas where tumor cells are likely to be more densely distributed contributes to a more accurate pathological diagnosis. This approach may also help prevent misdiagnosis as breast cancer, which can appear similar on imaging, thereby enabling timely and appropriate treatment for lymphoma.

## Data Availability

The data that support the findings of this study are available from the corresponding author upon reasonable request.
